# Implementation of a Work-Related Asthma Screening Questionnaire in Clinical Settings: Multimethods Study

**DOI:** 10.2196/37503

**Published:** 2022-09-15

**Authors:** Madison MacKinnon, Max Moloney, Emma Bullock, Alison Morra, Teresa To, Catherine Lemiere, M Diane Lougheed

**Affiliations:** 1 Asthma Research Unit Kingston Health Sciences Centre Kingston, ON Canada; 2 Division of Respirology Department of Medicine Queen's University Kingston, ON Canada; 3 The Hospital for Sick Children Dalla Lana School of Public Health University of Toronto Toronto, ON Canada; 4 Department of Chest Medicine Hôpital du Sacré-Cœur de Montréal Montreal, ON Canada; 5 Faculty of Medicine University of Montreal Montreal, ON Canada

**Keywords:** work-related asthma, asthma, dissemination, implementation, e-tools, barriers, limitations, electronic medical records, EMRs, knowledge translation, mobile phone

## Abstract

**Background:**

A work-related asthma (WRA) screening questionnaire is currently being validated for implementation in clinical settings. To minimize barriers to integrating tools into clinical practice, a discussion of strategies for the implementation of the questionnaire has begun.

**Objective:**

This study aimed to understand the benefits, feasibility, barriers, and limitations of implementing the Work-related Asthma Screening Questionnaire–Long version (WRASQ[L]) and asthma e-tools in clinical settings and propose dissemination and implementation strategies for the WRASQ(L).

**Methods:**

This study was conducted in Kingston, Ontario, Canada, from September 2019 to August 2021. A workshop and 2 questionnaires were used to understand the benefits of and barriers to implementing the questionnaire in clinical settings. An expert advisory committee was established to develop the implementation and dissemination strategies. Workshops were semistructured and used thematic qualitative analysis to identify themes that provided an understanding of the benefits and limitations of and barriers to using the WRASQ(L), and e-tools in general, in clinical settings. Workshop participants included patients and health care providers, including physicians, nurses, and asthma educators, who were implementation specialists and expert electronic medical record users. A questionnaire focusing on providers’ knowledge and awareness of WRA and another focusing on WRASQ(L) feedback was administered at the workshops. Advisory committee members from relevant stakeholders met 3 times to strategize implementation opportunities.

**Results:**

A total of 6 themes were identified in the workshop: involving and addressing patient needs, novel data collection, knowledge translation, time considerations, functional and practical barriers, and human limitations. Questionnaire responses yielded positive feedback on the utility of the WRASQ(L) in clinical settings. All participants agreed that it is an easy way of collecting information on occupational and exposure history and could prompt a discussion between the health care provider and patient on how the workplace and exposures could affect one’s asthma, increase awareness of WRA in patients and providers, and increase awareness of exposures in the workplace. Implementation and dissemination strategies were generated with input from the advisory committee.

**Conclusions:**

Stakeholders and workshop participants consider the WRASQ(L) to be a useful tool that satisfies many provider needs in their clinical settings. Once validated, dissemination strategies will include developing educational materials that include the WRASQ(L), linking the questionnaire to stakeholder websites or e-toolkits, translation into other languages, leveraging health care and research networks, conference presentations, and peer-reviewed publications. Implementation strategies will include integration into electronic medical records; designing multifaceted interventions; and targeting nontraditional settings such as workplaces, pharmacies, and research settings. The WRASQ(L) addresses many benefits of and barriers to implementation, as identified in the workshop themes. These themes will guide future implementation and dissemination strategies, noting that human limitations identified in providers and patients will need to be overcome for successful implementation.

## Introduction

### Background of Work-Related Asthma and Knowledge Translation

Work-related asthma (WRA) is identified as asthma that is exacerbated or caused by workplace exposure and is estimated to affect as many as 25% of adults with asthma [1]. The most effective way of diagnosing WRA is through a detailed occupational history and objective measurement of lung function [2,3]. However, physicians, particularly at the primary care level, often do not have the time and resources to take a detailed occupational history, and objective measures are expensive, time consuming, and often only available in specialized centers [3,4]. This gap in screening and awareness of WRA is believed to contribute to an average of 4 years of delay between symptom onset and diagnosis, which is associated with increased morbidity [4,5]. The diagnosis of WRA has been shown to improve patient outcomes, including health service use [6].

**Figure 1:**
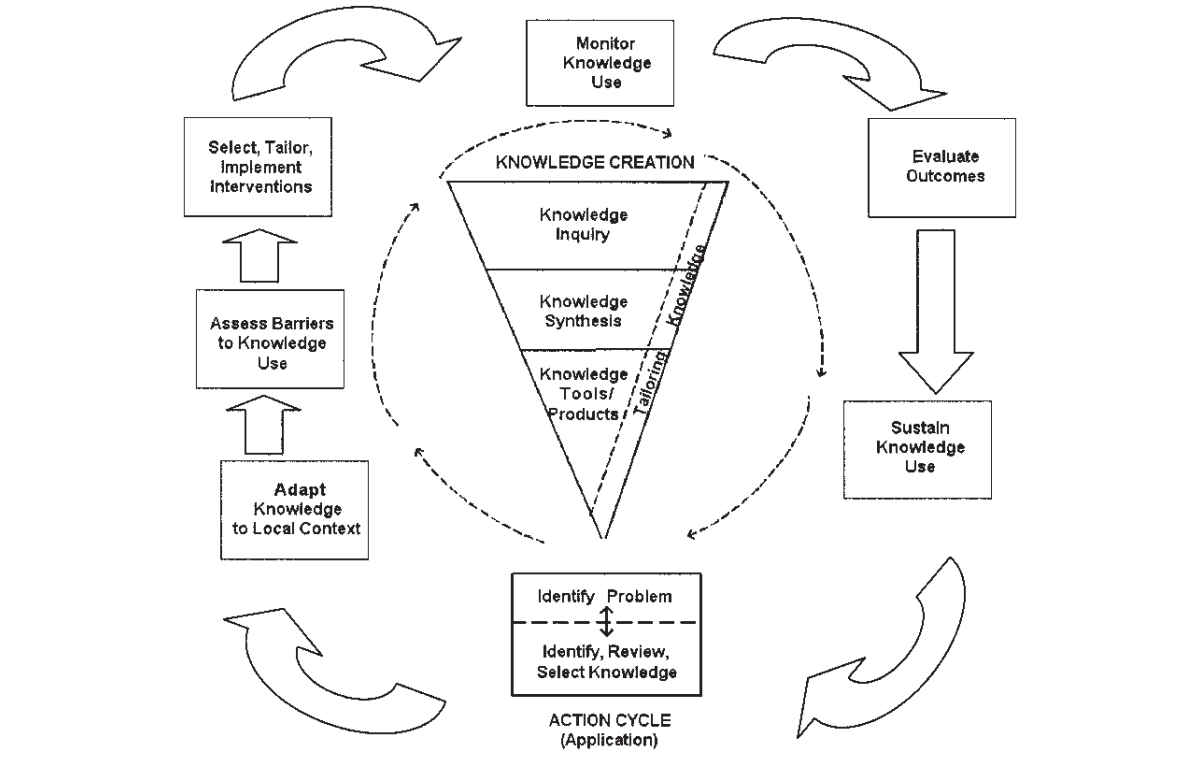
The knowledge-to-action process created and reproduced from Graham et al [7], which is published under CC-BY-SA license.

The knowledge-to-action (KTA) framework created by Graham et al [7] (Figure 1) outlines the elements involved in the KTA process to facilitate the implementation of research findings. The KTA framework is split into 2 dynamic concepts, called knowledge creation and knowledge action, each with its own respective phases [7]. Knowledge creation identifies the different types of knowledge and research available, and the knowledge action cycle identifies the pathway and steps to implementation, which, briefly, includes identifying a problem, adapting local knowledge to a particular context, assessing barriers, implementation, monitoring and evaluating interventions, and sustaining knowledge use [7].

In an effort to improve the accessibility, quality, and efficiency of the Canadian health care system, the Government of Canada has invested in eHealth [8]. eHealth describes the use of information and communication technology in health care and includes a wide range of technologies, including electronic patient records, telemedicine, chronic disease–monitoring systems and management tools, electronic prescribing, and decision support tools [8-10]. In general, eHealth tools have been found to reduce symptoms, improve self-management, improve patient-provider communication, and improve overall clinical outcomes [11]. Barriers to the implementation of health tools include poor accessibility, conflicts with a practice or the practice setting, financial incentives, individual beliefs and characteristics of health care professionals, and patient factors [12].

Integrated knowledge translation is defined as the knowledge exchange and collaboration between researchers and end users of research (eg, providers, patients, and policy makers) [13,14]. This process sees end users as partners in the research study and ensures that the asked research questions are of importance to end users rather than researchers [13,14]. End user involvement can vary widely among studies. A review examined the involvement of users in designing and evaluating self-monitoring applications for bipolar disorder. Across these studies, end user involvement ranged from just the evaluation stage of the application to all stages of research such as the evaluation, prototype design, and the concept of the application generation phases [15]. The involvement of stakeholders in the design process of interventions has been considered the “holy grail” for improvement and has been found to develop the capacity of researchers and decision-makers to engage in integrated knowledge translation processes and enhance the value of research for decision-makers [16,17]. However, few studies have included integrated knowledge translation strategies in the health care sector, and there are no clear guidelines or methodologies for end user involvement in research [16,17].

Current implementation and knowledge translation strategies for WRA, and asthma in general, have focused on the management and education of patients after their diagnosis [4]. These include prevention programs in high-risk industries [18-20], tools for patients to self-report asthma symptoms [21], and self-management plans in the form of digital applications or electronic books [22-24]. Many tools for clinicians have focused on disseminating guidelines into clinical practice to help with asthma management [4]. These include asthma care maps at the primary care level [25] and asthma care pathways in the emergency department [26]. There is a paucity of strategies or tools that focus on increasing awareness of potential WRA in individuals with asthma and few strategies or tools that are targeted to providers to increase awareness and screening of WRA [4].

### Background of the Work-Related Asthma Screening Questionnaire

The Work-related Asthma Screening Questionnaire–Long version (WRASQ[L]) was designed for implementation in clinical settings, particularly primary care, as a way of increasing awareness of WRA and screening for suspected WRA cases [27,28]. The WRASQ(L) collects occupational and exposure history, information on workplace-asthma symptom relationships, and personal protective equipment use. It includes an interpretation guide to prompt the provider on what steps to take if the patient screens positive for suspected WRA. The WRASQ(L) is available as a paper PDF, and it can be accessed through a patient or provider web-based portal via a kiosk or tablet in the waiting room or through a fillable PDF file linked to our hospital’s electronic medical record (EMR).

During its development, the WRASQ(L) was found to have good content and face validity, good test-retest reliability, and low respondent burden [28]. Although its final validation is underway, our research team has begun to strategize the implementation and dissemination opportunities for the questionnaire. We also aimed to understand the perspectives of providers and patients on the current gaps in asthma and WRA screening and management and their perspectives on the implementation of e-tools, particularly the WRASQ(L), into clinical settings.

### Purpose and Objectives

The purpose of this paper was to report the dissemination and implementation strategies we brainstormed to maximize the impact of the research findings of the WRASQ(L)’s final validation. Using workshops, questionnaires, and an expert advisory committee, we aimed to understand the benefits, feasibility, and limitations of and barriers to implementing the WRASQ(L), and asthma e-tools in general, in clinical settings, as seen by both patients and providers, to provide valuable insights into the most effective and efficient way of implementing the screening questionnaire.

## Methods

This study was conducted in Kingston, Ontario, Canada, from September 2019 to August 2021. The participants in the project advisory committee (PAC) were from the provinces of Ontario and Manitoba. The workshop participants were all from Ontario.

### PAC Role and Engagement

Although the final validation of the WRASQ(L) is underway, a PAC was formed to oversee the questionnaire’s final validation and strategize how to implement and disseminate the questionnaire once validated. The committee was engaged at the outset, during, and at the conclusion of the validation of the questionnaire to identify the integrated and end-of-grant strategies. Stakeholders from relevant groups were invited to participate, including but not limited to Health Canada; compensation boards; professional societies; provincial and national lung, asthma, and allergy associations; public agencies; nonprofit organizations; and research groups. Potential stakeholders were invited to join the committee via email. Terms of reference were developed, and the committee met approximately biannually from 2019 to 2021 for 1 hour. The first and seventh authors cochaired the committee and led the meetings. A total of 14 members joined, including physicians, researchers, asthma educators, and nurses, from Ontario and Manitoba. A project update on the validation of the questionnaire and current knowledge translation initiatives was presented at the beginning of each meeting, after which it was opened to discussion among members. Each member was given an opportunity to provide feedback on current knowledge translation initiatives and suggestions for other end-of-grant dissemination or implementation strategies. Meeting minutes were recorded and sent to members after each meeting. Summaries of the meetings and strategies are presented.

### Asthma e-Tools Workshop and Questionnaires

We conducted 2 web-based workshops to understand health care providers’ and patients’ perspectives on the benefits and limitations of and barriers to using e-tools, including the WRASQ(L), in clinical settings. The first workshop aimed to understand how to best integrate an asthma surveillance system, asthma indicators, and clinical guidelines in general into primary care EMRs. The second workshop focused on asthma e-tools developed by the Asthma Research Unit (ARU), including the WRASQ(L), with the aim of understanding the benefits and limitations of and barriers to using these e-tools in clinical settings. During the second workshop, there were presentations and demonstrations of the ARU’s e-tools, and participants were familiarized with WRASQ(L)’s purpose, formats, and goals for implementation.

Each workshop lasted 2 hours and was conducted via Microsoft Teams. Workshops followed a semistructured question guide (Multimedia Appendices 1 and 2). The question guide was separated into different sections, each with its own individual topic or topics of discussion. Each section was allotted a specific amount of time during the 2 hours to ensure that all topics were discussed. Workshops were led by a skilled moderator—a family physician from OntarioMD who was not an asthma expert. Each participant was encouraged to speak, and the moderator moved on to the next question only once the participants had nothing more to contribute. A notetaker was also present, and the entire workshop was recorded.

The first workshop included 7 attendees. A total of 6 attendees were selected by the organization (OntarioMD) that facilitated the workshop because of their expertise in EMR use and implementation. Of the 6 attendees, 5 (83%) attendees were family physicians, and 1 (17%) was a nurse practitioner. Our research team recommended that another family physician with a special interest in respiratory health participate as a guest expert. The second workshop contained 6 participants, of whom 4 (67%) were selected by OntarioMD, 2 (33%) were family physicians, 1 (17%) was a nurse practitioner, and 1 (17%) was a patient. Our team recommended that the same family physician and nurse practitioner who was also an asthma educator participate. The practitioners were all based in Ontario and had a wide range of locations of practice, from rural to urban centers. The guest participants added by our research team were purposeful and had extensive knowledge of asthma e-tools and EMRs in Ontario. Some participants knew the researchers and were familiar with previous work, whereas others were unfamiliar.

Questionnaires were sent to the participants before and after the second workshop (Multimedia Appendices 3 and 4). The first questionnaire, which was sent before the workshop, aimed to assess the providers’ knowledge and awareness of WRA. Providers were first asked whether they discussed occupational history with patients with suspected or confirmed asthma. If so, providers were asked whether this information was recorded (in EMRs, paper charts, or not at all). If this information was not recorded, providers were asked to provide a reason for not recording it. Subsequently, providers were asked whether they discussed potential WRA, workplace exposures, and the potential relationship between workplace exposures and asthma symptoms in the workplace with their patients. The second questionnaire, which was sent after the workshop, asked for specific feedback on the WRASQ(L). One of the questions used a Likert scale to understand how much participants agreed with statements about the WRASQ(L)’s utility in prompting a discussion on the relationship between workplace exposures and asthma, raising awareness of WRA and potentially harmful exposures, collecting occupational history, improving screening, and increasing referral time to a specialist for WRA. We then asked whether providers would consider administering the WRASQ(L) in their practice and, if so, asked for which purpose (screening for WRA, collecting occupational history, collecting information about the relationship between the patient’s asthma symptoms and workplace exposures, initiate a conversation about the topic of workplace-symptom relationship with patients, or others) and in what format.

The questionnaire responses were tallied by frequency and percentage. A total of 3 team members from the ARU participated in the thematic qualitative analysis of the workshops [29]. All the team members engaged in reflexivity throughout the research process [30]. The team members met at the beginning of the data analysis to discuss and record how their personal experiences and biases could influence their interpretation of the results. All were a part of the ARU and familiar with the e-tools, literature related to WRA diagnosis, and reporting and implementation of asthma e-tools. One of the team members took notes at all research team meetings, and all members recorded notes in a memo throughout the analysis.

The workshop audio was transcribed verbatim and rechecked by a different team member. All team members reviewed the transcripts multiple times, rewatched the recorded workshops, and reviewed their notes taken during the workshops to familiarize themselves with and obtain a general and descriptive sense of the data.

All members were engaged in the coding of the data. Coding was conducted separately, followed by regular team meetings to discuss codes, overarching themes, and impressions from the data. Codes were added to a codebook that was refined and narrowed down with subsequent coding sessions. Transcripts were reviewed and recorded until they agreed that data saturation had been met.

Relationships between codes were identified using tables and mind maps to organize the codes into overarching preliminary themes. Themes were reviewed over multiple group meetings with input from all team members and refined until the key themes that defined the essence of the data were agreed upon. The key themes were named and defined, and quotations that provided sufficient evidence of the themes were selected. Any disagreement during the coding or identification of themes was discussed and resolved by the group.

### Ethics Approval

The study was reviewed for ethical compliance by the Queen’s University Health Sciences and Affiliated Teaching Hospitals Research Ethics Board (approval numbers: TRAQ# 6029444 and 6019013).

## Results

### PAC Strategies

A total of 2 members affiliated with the Workplace Safety and Insurance Board Champions Program, a program working to implement occupational health modules in Ontario medical schools, suggested discussing the WRASQ(L) with a Queen’s University representative. There was also a discussion of partnering with the Lung Health Foundation as they are creating an e-module for providers on WRA. The PAC noted that translation into other languages should be considered, particularly in Chinese, as asthma is prevalent in the Asian community. They also suggested using the WRASQ(L) as a validated tool for research and as a way of placing occupational information that is clinically useful and relevant into EMRs, as 1 member mentioned that the Ministry of Labor was working on such an initiative.

One of the members noted that the European Respiratory Society Task Force was examining validated questionnaires to be used clinically and in research for WRA surveillance. Other health care provider networks were suggested to be leveraged, such as patient advocacy through the Lung Health Foundation and certified respiratory educators through the Canadian Network for Respiratory Care. A final suggestion was to consider implementing the WRASQ(L) in pharmacy settings for patients with suspected WRA who are yet to see a physician.

### Questionnaires

The questionnaire asking providers about their awareness of WRA was provided to those participants of the second workshop who were health care practitioners. Therefore, it was filled out by 83% (5/6) of participants. The questionnaire had a response rate of 100%. All participants (5/5, 100%) said they discussed their occupational history with patients with suspected or confirmed asthma, and most (4/5, 80%) stated that they recorded their occupational history in their EMR. One of the participants said that they asked the patient whether they wanted the detailed work history recorded or whether they just wanted an overview of it but did not specify where they placed it. Approximately 80% (4/5) of participants reported that they routinely discussed the potential relationship between workplace and asthma symptoms with patients with suspected or confirmed asthma. Approximately 20% (1/5) of participants said whether they are going to discuss depends on the age and stage of their asthma. All participants (5/5, 100%) reported inquiring about the exposures with which patients were in contact at their workplace in those with suspected and confirmed asthma. Finally, all participants except 20% (1/5) said that they discussed the management of asthma in relation to the workplace with patients with confirmed or suspected asthma.

The questionnaire that asked for WRASQ(L) feedback had a response rate of 80%. Overall, participants felt it was beneficial and could prompt a discussion between the health care provider and patient on how the workplace and exposures could affect one’s asthma, increase awareness of WRA in patients and providers, and increase awareness of exposures in the workplace. All participants strongly agreed that it was an easy way of collecting information on occupational and exposure history. Approximately 75% (3/4) of participants strongly agreed that the WRASQ(L) could improve the screening of WRA at the primary care level, speed the time to referral to a specialist, and decrease the time to diagnosis.

### Asthma e-Tool Workshops

#### Overview

A total of 6 themes explained health care provider preferences regarding the use of e-tools, particularly the WRASQ(L), in clinical settings, with subthemes that organize the narrative. These themes can be categorized into 3 benefits, 2 key barriers or limitations, and 1 considered both a benefit and a barrier or limitation. The themes were as follows: (1) involve and address patient needs, (2) novel data collection, (3) knowledge translation, (4) time considerations, (5) functional or practical barriers, and (6) human limitations (Table 1).

**Table 1:** Brief description of themes.

Theme	Benefit or barrier	Description
Involve and address patient needs	Benefit	It is important for patients to feel involved in their care, and thus, tools should enable this. This can be done by having flexibility regarding when the tool can be used and the format of the tool by providing feedback to patients and considering their fatigue and fears.
Novel data collection	Benefit	Tools should fill a gap in data collection or provide a unique way of collecting data.
Knowledge translation	Benefit	Tools are beneficial when they translate knowledge from the specialist to the primary care provider or the provider to the patient.
Time considerations	Benefit and barrier	Any tool that saves the provider time is incredibly beneficial; however, if it takes too much time to learn, use, and implement, it is a barrier.
Functional and practical barriers	Barrier	Limitations in technology, particularly seamless integration of tools into electronic medical records and for patient use, and resources will impede the use of the tool in practice.
Human limitations	Barrier	Provider and patient attitudes and behaviors, such as mistrust of tools and personal biases and fears, and the tendency to not reuse tools by stakeholders are all human limitations to the uptake of tools.

#### Involve and Address Patient Needs

The participants noted that e-tools that involve or inform patients about their care are beneficial to clinical practice. Patients take pride in and give importance to being involved in their care and seeing themselves as “partners” with providers in their care. Tools are not considered useful to the patient population if there is “no clear follow-up,” and a feedback loop to inform patients is beneficial:

I think the idea of the feedback loop is [a] really important one...Because many times we just collect data and we don’t actually let you know, or to what end, or to let you give any sort of response on to what we’ve done as a result of that. So, I think that there’s potential for the applications or these tools to do that in real-time.

Flexibility in when patients use e-tools or the format in which they are administered has been frequently discussed. Questionnaire fatigue was mentioned, with concern over how fatigue can affect the authenticity or accuracy of the answers. Another concern was the mistrusted answers from patients who complete questionnaires in stressful situations as they are just trying to get the questionnaire “out of the way.”

In discussing potential solutions, a popular option from both patient and physician peer leaders was flexibility, both in the timing and format of the questionnaire or e-tool administration. The options discussed were before visits, in the waiting room, or during the visit while the provider was doing other clinical activities, ultimately wherever made the patient most comfortable:

...in the waiting room I would love to have something to fill out...It is the perfect opportunity. I think the way I would prefer it to happen would [be] to get an email a couple days before an appointment and have the opportunity to fill out but if I don’t, then I’m handed a tablet at the appointment visit to fill it out right. I think you gotta use both strategies, not one or the other.

#### Novel Data Collection

Tools must fill a certain gap in data collection or provide a unique aspect to data collection to be considered beneficial by providers. In the workshops, participants mentioned the underreporting and undermanagement of asthma in the population. For example, they found it difficult to document occupational history in the EMRs. A tool that fills these gaps and provides an opportunity for these data to be recorded would provide a major benefit to clinical practice.

Tools that present data in a unique manner, such as through visualizations of the data, benefit practice. The asthma educator participant emphasized that tools that can show novel trends in the data for patients are very beneficial:

I’ve had patients say it is really helpful to see how that tool is able to give me a visual on how this has improved my life...

#### Knowledge Translation

Tools must facilitate the translation of knowledge from specialists to generalists or from providers to patients. Participants noted this comes from a clear understanding of what the tool does and how to use it and, ultimately, how to use it to improve their care:

I think that part of it needs to be solved just in...the knowledge translation, what is this tool actually for

The providers discussed that the integration of the tools into clinical settings is an incredibly important step in the knowledge translation process. It facilitates the movement of information to the provider or patient and helps physicians and patients “manage their issues in the most optimal way.”

#### Time Considerations

Time was a central theme in both workshops, particularly for providers. Participants emphasized that tools with time-saving features were incredibly helpful and more likely to be used. Automated features, such as drop-down menus or a proactive reminder to use the tool, were viewed favorably by the participants. Participants emphasized that tools should be efficient and easy to use so that they do not affect their practice:

We have to make it as easy as possible; I think that’s kind of the key...otherwise people are not going to do it.

Conversely, if a tool takes too much time to learn or use, then it is a major limitation or barrier to using the tool. One of the participants noted that time constraints in the clinic could prevent them from using the tool, even if it was already implemented.

#### Practical and Functional Limitations

One of the main limitations discussed by the participants was practical or functional limitations, in other words, a lack of resources and technological limitations in accessing, using, and implementing e-tools. Almost all participants emphasized that tools need to be seamlessly linked or integrated into the EMR. A seamless linkage in practice and in using e-tools is considered when the provider does not have to leave their EMR or patient charts to access e-tools or other programs. It also includes the transfer of data from the tool or program back to patient charts. Many participants said that having to leave their EMR to use a tool is a major barrier, and the interconnectedness of tools is currently lacking:

I meant the integration itself is just so important...I mean, beyond just leaving the environment you’re in, which would be a real pain and is certainly a barrier to adopting these things, but like the integration piece allows you to bring data in...But nobody’s really figured this out or I don’t know of anybody that’s figured this out...

Providers frequently noted that a lack of physical, human, technological, and financial resources are major barriers to implementation. Managing the data that comes from these tools was noted to be difficult if there were not as many resources available, causing more work for the provider, which may ultimately stop providers from using the tool.

#### Human Limitations

##### Provider Behavior

Providers’ behaviors and preconceived ideas or biases are major limitations to the use and implementation of e-tools. Fear and apprehension about how e-tools could negatively affect providers were mentioned many times:

I think on the other side there’s always this fear that the data is somehow going to be used for, you know, negotiations or if it’s in the wrong hands is going to be used against the physician in some sort of way which, which is obviously far from the truth.

Participants corroborated this from their own experiences when they implemented their own e-tools in the form of a dashboard. They noted that stakeholders asked how the information would be used to “punish” them, and there was a “suspicion” they were sharing the data.

Convincing providers to try a new tool was another barrier. It was considered difficult to market a new tool to providers, and providers were noted as having a “defeatist attitude” that new e-tools are “not useful at all” when they do not work perfectly, meet the providers’ expectations immediately, or are slow to be implemented. Behavior change is required for providers to adopt and implement a new tool. Incentives, such as funding, and quick turnaround of information have been mentioned as ways of inciting behavior change.

##### Trust and Proof of Value

Trust must be established between the tool and the patient or provider. The user needs to feel that they can trust the tool and the information provided and that it will make a difference in their practice. Trust was established by determining whether the provided data were accurate. One of the participants mentioned that when discussing new e-tools or implementations with providers, “the immediate discussion goes to ‘well that’s not accurate.’” Providers prioritize and need to see clear and accurate data to adopt a new tool as this is proof that the tool will be valuable to them. If providers and patients do not see the value of the tool, then it is unlikely to be reused. Both provider and patient participants expressed that tools or apps can be forgotten if not deemed useful:

I use a lot of apps in my practice on my mobile devices and the good ones I use regularly and the ones that aren’t that great you stop using.

If patients and providers can see how it improves practice, it will establish a level of trust and change behavior. Seeing and understanding the proof of value of the tool can lead to the desired outcome of sustained use of the tool in practice. Overcoming alert fatigue, which was mentioned twice by participants, and “rewarding good outcomes and good behaviour” are the means to achieve this outcome.

## Discussion

### Principal Findings

We aimed to understand the benefits, feasibility, limitations of and barriers to implementing asthma e-tools in general and, specifically, the WRASQ(L), in clinical settings, as seen by both patients and providers. Through the focus groups and PAC, we gained information from both end users and specialists in the field. Our findings provide insights into the potential implementation and dissemination opportunities for WRASQ(L). Our findings suggest that the questionnaire, and e-tools in general, are considered useful in clinical settings and have the potential to greatly improve practice. The identified key barriers need to be overcome to facilitate the adoption of asthma e-tools.

Through the preworkshop survey, we found that workshop participants reported discussing relevant information with their patients who had suspected and confirmed asthma, such as symptom-workplace relationships and management and recording occupational history. These results contrast with those found in the literature, which identifies a major gap in care in primary care settings for WRA, especially in taking detailed occupational history, workplace exposure history, or discussion on how asthma might be work related [4,31]. Our results may differ because of the selection bias of the participants. Some participants had a keen interest in respiratory health and were expert users of EMRs; hence, they may ask for and record this information in their practice. The postworkshop questionnaire results were encouraging; all participants agreed that the WRASQ(L)’s implementation would be beneficial, that it could speed up the time to referrals to a specialist to ultimately decrease the delays in diagnosis, and that it was a useful tool to collect occupational history.

Our workshop concentrated on the benefits and limitations of and barriers to implementing e-tools in clinical practice. We used an inductive approach to understand the health care provider and patient perspectives on e-tools [29]. The themes pertained to involving patients in their care, creating a new type of data collection, facilitating knowledge translation, saving and not taking up too much time, and overcoming functional barriers and human limitations. The findings suggest that, overall, e-tools are considered beneficial in clinical settings but only if their implementation and use can overcome the identified barriers. The findings also provide important context and knowledge on how to best implement WRASQ(L) and ensure the future use of the tool.

**Figure 2:**
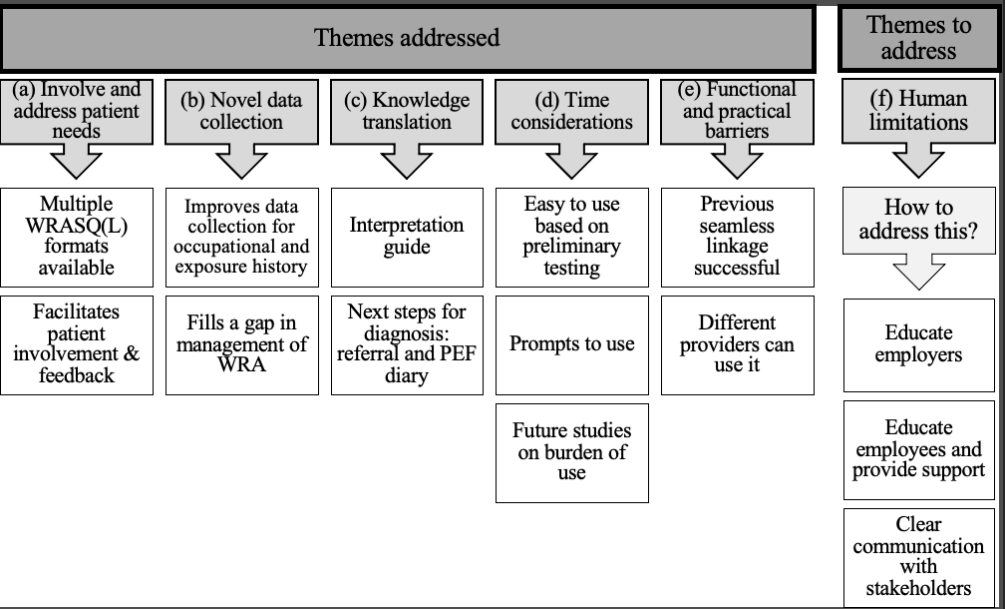
Themes addressed and themes to address by the WRASQ(L). PEF: peak expiratory flow; WRA: work-related asthma; WRASQ(L): Work-Related Asthma Screening Questionnaire–Long version.

Although many of the themes identified in our workshops have been reported in the literature, the WRASQ(L) addresses and expands on these themes in the context of implementing a WRA tool in primary care EMRs (Figure 2). The workshop results also established a novel theme of knowledge translation. It was established that patients take pride in their care, want to be involved in their care, and want feedback. This has been noted in the literature, particularly in studies involving patients with chronic diseases [32,33]. A benefit of the WRASQ(L) is its ability to easily collect occupational history and its many different forms. It can be filled out on paper, electronically, or via a kiosk that is accessed from a portal via a smartphone or tablet. This allows flexibility in how patients complete the WRASQ(L). If completed in the clinic, the questionnaire can be interpreted immediately, which would provide immediate feedback to the patient. These benefits address the themes of “Involve and Address Patient Needs” and “Novel Data Collection.” The WRAQS(L) addresses questionnaire fatigue as well, as it has a low respondent burden and takes, on average, <10 minutes to complete (mean 7.2, SD 3.8 minutes), which makes it a timely questionnaire to complete [28].

Time constraints have been stated as the most important barrier to taking an occupational history; therefore, this theme emulates one of the key barriers to diagnosing WRA [4,31]. Our participants showed enthusiasm for automatic features, and it has been found that successful implementation strategies previously included the use of reminders for the tool [12]. Although the WRASQ(L) does not have reminders, it has easy-to-fill options and a prompt to fill out the questionnaire in the asthma management systems to which it is seamlessly linked. The literature, and workshop findings, suggest that implementation should occur within a realistic time frame and that the clinical utility of tools is maximized when the tool is time efficient and easy to use [12,34]. The WRASQ(L) has been found to have a low respondent burden and good test-retest ability; therefore, it has been deemed easy to administer. No studies have examined the burden of using the questionnaire in clinics; however, this leads to future studies after implementation. Our participants greatly emphasized the vertical integration of tools into EMRs. The WRASQ(L) has already been successfully integrated into an asthma management system that seamlessly links to a fillable PDF file and kiosk version.

An interesting result was that providers and patients themselves could be barriers to adoption. Providers’ preconceived fears, notions, and attitudes regarding the implementation and use of new tools were very evident. Behavior, or more specifically, attitude change, is very important for implementation as it is unlikely that the tool will be implemented if the provider is not receptive to change or their concerns are not addressed [12]. Therefore, although human limitations are a general barrier to implementation reported in the literature, researchers should identify the specific human limitations that relate to the conditions they are studying, which could affect their implementation of the e-tool. Behaviors by providers and participants specific to occupational diseases are foreseeable barriers to implementing our questionnaire. Providers hesitate to diagnose and manage occupational diseases because of the burden of submitting a compensation claim [35,36]. Patients avoid discussing their health concerns in the workplace for fear of the stigma associated with injured workers; in particular, fear that coworkers or managers will think they are abusing the system or malingering [35,37]. There is a hesitancy to file claims or report health issues, as well as the fear of losing their jobs [4,35,38]. Thus, we need to address these specific concerns regarding human behaviors in work-related conditions when moving forward with our implementation. In addition, this theme showed that clear communication about the purpose of the tool is needed, and providers need to feel that they understand and trust the tool. This is established by longevity; if patients or providers continually use the tool, then this is the desired outcome. Ultimately, sustained use of the tool in clinical settings is the overall goal.

A novel theme identified by our focus groups was that it would be ideal if a tool could be a conduit for knowledge translation; that is, tools were considered beneficial when they provided knowledge exchange between providers and from providers to patients. This is particularly beneficial for an underreported disease such as WRA, and we believe that the WRASQ(L) is able to address this. The WRASQ(L)’s interpretation guide outlines the recommended steps in the care of the patient; thus, it conducts knowledge translation by bringing this expert knowledge from specialists to the primary care level and the patients. In addition, by simply using the WRASQ(L) once, patients and providers are made aware of the potential harmful exposures that could cause WRA, the relationship between asthma symptoms and the workplace that could indicate WRA, and the importance of taking an occupational history. Thus, the creation and implementation of e-tools should prioritize an element of knowledge transfer between users and the literature for ideal and long-term implementation.

Our workshops identified how the WRASQ(L) could benefit clinical practice from the viewpoint of end users. Once limitations are addressed, the strategies from the specialists in the PAC can be used to implement the questionnaire in the field. Discussions from our PAC focused on leveraging and connecting with other health care networks and stakeholders with whom many members were affiliated or had worked with before. Ultimately, we proposed several strategies for use once the final validation of the WRASQ(L) was completed (Textbox 1).

**Summary of implementation and dissemination strategies.**

**Implementation strategies**

- Integrate into electronic medical records
  OntarioMD’s dashboard
  Collaborate with electronic medical record vendors, Ministry of Labor, and Ministry of Health
- Design multifaceted interventions
  Prompts and reminders
  Performance indicators
  Audit and feedback
- Target nontraditional settings
  Workplaces
  Pharmacies
- Implement in research settings as well as clinical

**Dissemination strategies**

- Develop educational material
  Workplace Safety and Insurance Board Champions Program
  Lung Health Foundation’s e-module for providers on work-related asthma
- Link to websites or electronic toolkits
- Lung Health Foundation’s “current educational strategies” for providers
- Canadian Thoracic Society toolkit
- Translate to other languages
- Leveraging existing health care and research networks:
  Certified respiratory educators: Canadian Network for Respiratory Care and Primary Care Asthma Program
  Canadian Thoracic Society Asthma Clinical Assembly
  American Thoracic Society and European Respiratory Society Work-Related Asthma Taskforces
  Center for Research Expertise in Occupational Disease
- Conference presentations
- Peer-reviewed publication

The dissemination strategies proposed by the PAC included common actions such as conference presentations and peer-review publications; however, members also suggested implementing the WRASQ(L) as educational material and linking it to websites or e-toolkits. These strategies have the potential to have a 2-fold effect. They could not only increase the use of the WRASQ(L) by providers but could also increase awareness of WRA. This would address a major concern with WRA by addressing the paucity of strategies or tools that focus on increasing awareness and screening for potential WRA among providers [4]. The translation of the questionnaire into other languages would further contribute to this. This would allow the administration of the questionnaire to populations at a higher risk of asthma, such as many Asian communities. Furthermore, it would increase the accessibility of the questionnaire to other providers in Canada and around the world, thus, potentially increasing the use of the questionnaire and increasing awareness of WRA in these places. Finally, the PAC members noted that other health care networks could be included more frequently in the dissemination process. Certified respiratory educators were particularly noted as a key network to leverage as they are imperative to the education of patients. In addition, focusing efforts on leveraging other research networks such as the Centre for Research Expertise in Occupational Disease would provide the opportunity for the questionnaire to be used in other research settings (a noted implementation strategy discussed in the following paragraph) and to be seen by other researchers, which has the potential to increase the use and awareness of the questionnaire.

Implementation strategies were more general than dissemination strategies but still provided guidance on how to increase the use of the questionnaire and awareness of WRA. Targeting nontraditional settings such as workplaces, pharmacies, and research settings has the potential to increase awareness of WRA in these places when it might be lacking. For example, placing the questionnaire in a workplace that has a high risk of WRA could inform workers and employers of the potential for WRA in the setting. This would not only create awareness of the issue but could also prompt employers to be aware of their workers’ conditions, mitigate risk with the provision of personal protective equipment, increase communication of the potential for WRA between employers and employees, and decrease stigma or fear of reporting WRA. Similarly, implementing the questionnaire in pharmacies and research settings would make other health care professionals such as pharmacists and other researchers more aware of WRA, despite not using the questionnaire for clinical purposes. Integration into EMRs was a concrete strategy that was proposed, and partnering with existing dashboards such as OntarioMD’s Insights4Care dashboard would allow for easy implementation. As stated, one of the members suggested that the Ministry of Labor was working on a way of including clinically useful occupational information in EMRs. Approaching bodies such as this early in their implementation process would ensure the WRASQ(L) is included as well. As implementation is a lengthy process, it is wise to explore these options so that implementation and dissemination can be timely once the questionnaire is validated. To the best of our knowledge, this method of approaching end users through our workshops and experts via the PAC before the actual implementation of the questionnaire is novel. Obtaining this knowledge will not only allow the implementation to be timely but also create and guide a robust implementation strategy for the questionnaire to maximize its use.

Our findings address both the action cycle and knowledge creation concepts in the KTA framework. Each phase of the knowledge creation concept allows researchers to tailor their activities to the needs of their ideal stakeholders and customize their methods of dissemination [7]. The workshop provided valuable insights into how we can tailor the WRASQ(L) to satisfy the concerns of stakeholders (patients and providers). Both the PAC and workshop findings addressed the steps “Adapt Knowledge to Local Context” and “Assess Barriers and Knowledge Use” in the action cycle. By reviewing current KTA knowledge and initiatives with the PAC and discussing gaps in management in the workshop, we adapted current knowledge to our context, which is the improvement of asthma management with e-tools. Many barriers were assessed by both groups. This will allow us to move confidently into the “Select, Tailor and Implement Interventions” phase to implement our concrete strategies while noting the barriers we may face.

A limitation of this study is that it does not include a theoretical framework for assessing the determinants of successful implementation of the questionnaire; however, we believe that the specific tool and context in which we aim to implement the tool benefited from an inductive approach. There is some concern that the use of frameworks can influence deductive analysis, bias researchers, and unconsciously force themes discovered into preconceived categories [39,40]. This was a potential concern because of the context of our study and our tool. WRA is a subtype of asthma that is unfamiliar to many patients and providers [4,5], and we aimed to understand the use of a tool for this specific disease if implemented in EMRs in primary care. In addition, we are at a very preliminary stage in the implementation process, and to the best of our knowledge, a similar study has not been completed previously for WRA screening tools. Thus, an open-ended approach allowed us to gather as much information as possible from stakeholders about the benefits of and barriers to implementing the tool in this context. The identified barriers and themes, along with a theoretical framework, could guide a robust and efficient implementation strategy once the validation is complete.

Our methods and findings allow the research team to approach these ideas and address potential limitations and barriers early to ensure efficient and timely implementation of WRASQ(L). These methods may be applied to other studies that validate e-tools, particularly studies that consider the involvement of end users and experts to discuss implementation strategies before the completion of the validation.

### Limitations

This study has several limitations. The small sample size and lack of use of the theoretical framework in this study may have reduced the generalizability of the results. Selection bias was present in our participants, as 33% (2/6) of participants with a keen interest in respiratory health (a family physician and an asthma educator) were invited. This decision was made to address what we felt was a serious limitation in the peer leaders selected by OntarioMD, as they lacked practical primary care expertise in asthma. A total of 3 workshop participants were familiar with the WRASQ(L) and ARU e-tools. Despite this, we felt that their contribution to the focus groups was beneficial, as they brought practical primary care asthma expertise into the discussion. Of the 3 participants, 2 (67%) had no experience using the WRASQ(L) in clinical settings, nor had they been asked whether they would use the tool in their practice. One of the participants who had used the questionnaire before in clinical settings was considered an important contributor as they could provide a unique perspective of how patients responded to the tool. All 3 participants were expert EMR users; thus, their insight was valuable. The use of a third-party moderator for the workshops mitigated bias by ensuring that all participants had an equal chance of contributing to the discussion. Finally, only one-half of a workshop focused on the WRASQ(L), making it challenging to identify a clear overarching implementation strategy. This was offset by the PAC, whose sole purpose was to discuss the implementation and dissemination of the questionnaire; however, the PAC members were not implementation specialists. It may be beneficial to conduct another workshop for only the questionnaire with a larger sample size.

### Conclusions

By addressing both the knowledge action and knowledge creation phases in the KTA framework, we identified key strategies to support the implementation of the WRASQ(L). Participants perceived the high utility of this WRA screening questionnaire in clinical settings and that it addressed many themes identified in our workshops relating to the implementation of e-tools in primary care EMRs. The workshop results and PAC recommendations will guide future dissemination and implementation initiatives and may be generalizable to other asthma e-tools.

Dissemination strategies will include incorporating the questionnaire in educational material, linking the questionnaire to websites or e-toolkits, translating it into other languages, and leveraging health care and research networks. Implementation strategies will include the integration of the WRASQ(L) into EMRs; designing multifaceted interventions; and targeting nontraditional settings such as workplaces, pharmacies, and research settings. The theme or barrier of human limitations may require more time and effort to overcome once the implementation of the questionnaire begins.

## Appendix

Multimedia Appendix 1 Interview guide for workshop 1.

Multimedia Appendix 2 Interview guide for workshop 2.

Multimedia Appendix 3 Questionnaire assessing providers’ knowledge and awareness of work-related asthma.

Multimedia Appendix 4 Questionnaire for the Work-Related Asthma Screening Questionnaire–long version feedback.

## Abbreviations

ARU: Asthma Research Unit
EMR: electronic medical record
KTA: knowledge-to-action
PAC: project advisory committee
WRA: work-related asthma
WRASQ(L): Work-related Asthma Screening Questionnaire–Long version
