# Supplementary material for: Implementation of a Work-Related Asthma Screening Questionnaire in Clinical Settings: Multimethods Study
Source: JMIR Form Res. 2022 Sep 15;6(9):e37503. doi: 10.2196/37503 (PMC9523520; doi:10.2196/37503)
Supplement: Multimedia Appendix 1 [file formative_v6i9e37503_app1.pdf]

1.0 MEETING DETAILS			
**Date:**	March 23, 2021	**Time:**	2-4 PM
**Location:**	**Join on your computer or mobile app**<br>Click here to join the meeting<br>**Or call in (audio only)**<br>+1 647-749-9252, 128500833# Canada, Toronto<br>(844) 564-3024, 128500833# Canada (Toll-free)<br>Phone Conference ID: 128 500 833#		
**Background:**	The research team is exploring how to integrate asthma tools and clinical guidelines in electronic medical records (EMRs) across Ontario (and Canada). Potential areas of exploration include:<br>• Asthma indicators in OntarioMD's Insights4Care Dashboard initiative;<br>• Exploring opportunities to engage clinicians (and patients if desired) on the AsthmaLife.ca tool;<br>• Exploring opportunities with electronic medical records (EMRs) including the integration of guidelines into the care process, real-time performance feedback, surveillance, and benchmarking;<br><br>The goal of this first workshop is to utilize OntarioMD's Peer Leaders with experience with the i4C Dashboard to discuss potential OMD dashboard indicators for asthma and the notion of a primary care surveillance system for asthma to improve quality and patient outcomes.		

2.0 WELCOME AND INTRODUCTIONS (10 mins)

Hi Everyone – welcome to our session this afternoon. We very much appreciate you taking the time to join us today and really appreciate your perspectives. There are no right or wrong answers. We want to hear from everyone today and are not seeking consensus. We will be recording the session, but names will not be associated or attributed to any comments.

We will be using our 2 hours together to achieve a few things:

- Firstly, we will be discussing the notion of a primary care surveillance system for asthma to improve quality and patient outcomes and would like to hear your perspectives of whether this would be useful in Primary Care for physicians like yourselves.
- We will also be discussing a series of potential dashboard asthma indicators and if physicians would find these valuable in informing patient care and treatment.

So, let's get started.

3.0 MEETING DISCUSSION (110 mins)

Objective	Description
Questions for discussion on surveillance and reporting (30 mins)	This first portion of the focus group will aim to gain a better understanding of the value of a primary care surveillance system for asthma to improve quality of care and patient outcomes.<br><br>**Goal: Understand what an asthma surveillance system is, and how we might scale one to reach most primary care practices in Ontario (and Canada)**<br><br>1. Do you think there is value in creating a primary care surveillance system for primary care physicians?<br>2. Do you have any concerns with implementing a surveillance system for quality improvement? What are the challenges/limitations?<br>3. Which outcome or population reporting tools do you use most often? Is the i4c Dashboard applicable here? Could it be?

<table>
<tr><th colspan="2">3.0 MEETING DISCUSSION (110 mins)</th></tr>
<tr><th>Objective</th><th>Description</th></tr>
<tr><td></td><td>a. What are the pros and cons to these?
b. For OSCAR/Trinity Healthcare Technologies users (if applicable): How does this EMR’s dashboard compare to others you have used?
4. What do you believe are the greatest barriers to the scalability of electronic clinical decision support and reporting tools in clinical practice?

Goal: Learn how to provide feedback within an EMR that is easy for clinicians to use and which leads to sustained behaviour change
5. If receiving a report on your practice, which format would you prefer:
a. Embedded within dashboard; auto-filtered, disease-based population reports
b. Email
6. If receiving a report on your practice, what would be the ideal frequency?
a. Real-time
b. Bi-weekly
c. Monthly
d. Quarterly
e. Half yearly
f. Yearly
7. Which report styles do you prefer?</td></tr>
<tr><td>Review of potential dashboard asthma indicators (45 mins)</td><td>This portion of the focus group will aim to gain a better understanding of what physicians want to measure and which asthma indicators clinicians would find valuable in OntarioMD’s dashboard.

Refer to Excel spreadsheet for list of potential child and adolescent quality indicators.

1. Are you aware of asthma quality indicators? (eg. PC-API, HQO)
2. Do you use them? If so, how?
3. Are you aware of PRESTINE asthma elements for EMRs?
4. What do you want to measure in patients with asthma?
5. What indicators do you think are valuable to have in the dashboard for asthma?
6. Do you think there should be different indicators by age group? Children and adolescents versus adult
7. What do you believe are the most important gaps in asthma management in primary care? Can these be improved by a surveillance system? What indicators would enable that? (eg. Confirmation of diagnosis, adherence to medications, assessment of control, access to asthma education)</td></tr>
<tr><td>Indicators Survey (30 mins)</td><td>With the above in mind, review the Excel spreadsheet of asthma indicators and rate each based on the following:

• Strength of evidence
• Relevance
• Feasibility
• Overall score and rank</td></tr>
<tr><td>Closing Remarks/Advice (5 mins)</td><td>• Given today’s conversation, are there any other comments or advice that you would like to add?</td></tr>
</table>

3.0 MEETING DISCUSSION (110 mins)	
**Objective**	**Description**
	THANK YOU
