# Supplementary material for: Implementation of a Work-Related Asthma Screening Questionnaire in Clinical Settings: Multimethods Study
Source: JMIR Form Res. 2022 Sep 15;6(9):e37503. doi: 10.2196/37503 (PMC9523520; doi:10.2196/37503)
Supplement: Multimedia Appendix 2 [file formative_v6i9e37503_app2.pdf]

1.0 MEETING DETAILS			
**Date:**	March 26, 2021	**Time:**	1-3 PM
**Location:**	**Join on your computer or mobile app**<br>Click here to join the meeting<br>**Or call in (audio only)**<br>+1 647-749-9252, 455725215# Canada, Toronto<br>(844) 564-3024, 455725215# Canada (Toll-free)<br>Phone Conference ID: 455 725 215#		
**Background:**	The research team is exploring how to integrate asthma tools and clinical guidelines in electronic medical records (EMRs) across Ontario (and Canada). Potential areas of exploration include:<br>• Asthma indicators in OntarioMD's Insights4Care Dashboard initiative;<br>• Exploring opportunities to engage clinicians (and patients if desired) on the AsthmaLife.ca tool;<br>• Exploring opportunities with electronic medical records (EMRs) including the integration of guidelines into the care process, real-time performance feedback, surveillance, and benchmarking;<br><br>The goal of this first workshop is to utilize OntarioMD's Peer Leaders from the i4c working group to obtain peer leader input into how best to integrate asthma tools that support best practice into primary care EMRs, and what electronic tools would be most useful.		

2.0 WELCOME AND INTRODUCTIONS (10 mins)

Hi Everyone – welcome to our session this afternoon. We very much appreciate you taking the time to join us today and really appreciate your perspectives. There are no right or wrong answers. We want to hear from everyone today and are not seeking consensus. We will be recording the session, but names will not be associated or attributed to any comments.

We will be using our 2 hours together to achieve a few things:

- Firstly, we will be discussing how EMR tools can support best practices for asthma in primary care and demonstrating specific eTools that physicians may find valuable in the EMR.
- Secondly, we will describe a work-related asthma questionnaire and discuss if providers would find it useful in primary care, in either a paper or electronic format.

So, let's get started.

3.0 MEETING DISCUSSION (110 mins)

Objective	Description
Questions for discussion on eTools (70 mins)	This first portion of the focus group will aim to gain a better understanding of how EMR tools can support best practices for asthma in clinics to improve quality of care and patient outcomes.<br><br>**Goal: Understand how EMR tools can support best practice in clinic**<br>1. Do you use eTools in your clinic?<br>a. Which ones?<br>b. Are they patient tools? Or practice evaluation tools?<br>2. Are these 'stand alone or integrated into your EMR in any way?<br>3. In general, are new eTools best used when integrated into clinical care systems? What does integration look like in a dashboard/clinical practice? Do they need deep integration or simply launching from your point of care?<br>4. What type of eTools would you like to see used in primary care practices? Describe the best functionality for uptake and advanced use. How would these lead to practice change?

	**Goal: Determine which eTools to implement into the dashboard or EMR environment**<br>1. PowerPoint presentation on asthma eTools<br>2. Demo<br>3. Discussion: Patient Questions<br>a. Do you think patient questionnaires are useful, why?<br>b. Have you ever been asked to complete a patient questionnaire?<br>c. Where and how often are you asked to complete patient questionnaires?<br>d. When would you prefer to complete a questionnaire a) in the waiting room, b) during my patient visit, c) prior to my appointment, d) after my appointment?
Questions for discussion on work-related asthma (30 mins)	The second portion of the focus group will aim to gain a better understanding of potential utility of a work-related asthma screening questionnaire.<br><br>**Goal: Understand how to assess for WRA in primary care**<br>1. PowerPoint Presentation on the WRASQ(L)<br>2. Discussion<br>a. Is there value in incorporating WRA screening into clinical practice? Would use the WRASQ(L)?<br>b. Is it feasible to incorporate into clinical practice?<br>c. Are there any barriers you foresee in using it in clinical practice?<br>d. What is the best way to integrate it into practice and into EMRs?<br>e. Is WRA assessment or completion of the screening tool relevant for the dashboard?<br>f. Is there any other location/website where the WRASQ(L) could be accessed easily?
Closing Remarks/Advice (10 mins)	Given today's conversation, are there any other comments or advice that you would like to add?<br><br>**** PROVIDE LINKS TO THE 2 SURVEYS ****<br>THANK YOU
