# Supplementary material for: Implementation of a Work-Related Asthma Screening Questionnaire in Clinical Settings: Multimethods Study
Source: JMIR Form Res. 2022 Sep 15;6(9):e37503. doi: 10.2196/37503 (PMC9523520; doi:10.2196/37503)
Supplement: Multimedia Appendix 3 [file formative_v6i9e37503_app3.pdf]

1. Do you ask/discuss the occupational history of a patient who have suspected or confirmed asthma?
   A. Yes
      If yes, how do you record their occupational history (***select all that apply)***:
      - In patient charts (paper)
      - Electronic medical records
      - Only if the patient mentions their workplace
      - Just discuss it; I do not record anything
      - Other: ___________
   B. No
      If no, why not? (***select all that apply***)
      - Lack of time
      - Do not think to ask
      - Forget to ask
      - Other: __________
   C. N/A

2. With your patients who have suspected or confirmed asthma, do you:
   a. Discuss the potential relationship between the workplace and asthma symptoms? **(Yes, No, Other)**
      a. If No: Are you aware of the potential relationship of the workplace and one's asthma symptoms? (**Yes, No, Other)**
   b. Inquire about exposures they are in contact with at their workplace? **(Yes, No, Other)**
   c. Discuss management of asthma in relation to the workplace (ie how to avoid exposures, what personal protective equipment should be worn, etc.)? (**Yes, No, Other)**
   d. N/A
