# Supplementary material for: Implementation of a Work-Related Asthma Screening Questionnaire in Clinical Settings: Multimethods Study
Source: JMIR Form Res. 2022 Sep 15;6(9):e37503. doi: 10.2196/37503 (PMC9523520; doi:10.2196/37503)
Supplement: Multimedia Appendix 4 [file formative_v6i9e37503_app4.pdf]

1. Please rate how much you agree with the following statements. There is a space to provide a comment if you would like.
   (Likert scale: N/A, Strongly disagree, disagree, neither agree nor disagree, agree, strongly agree)
   (*Comment option after each statement*)

   a. The WRASQ(L) could prompt a discussion on how the workplace and its exposures can affect one's asthma between a patient and healthcare provider
   b. The WRASQ(L) could help increase awareness of WRA or the relationship between the workplace and asthma symptoms in healthcare providers
   c. The WRASQ(L) could help increase awareness of WRA or the relationship between the workplace and asthma symptoms in patients
   d. The WRASQ(L) could increase awareness of potential harmful exposures at the workplace
   e. The WRASQ(L) is an easy way to collect information on occupational history and workplace exposures.
   f. The WRASQ(L) could improve screening of WRA at the primary care level
   g. The WRASQ(L) could speed up the time to referral of a specialist and decrease the time to diagnosis of WRA

2. Would you consider using the WRASQ(L) in your practice? **(*Yes,No, N/A & space for comment*)**

If Yes, then:

3. To whom would you consider administering the WRASQ(L) **(*select all that apply*)**:
   a. All patients?
   b. Only patients with confirmed asthma?
   c. Those with suspected asthma that has yet to be confirmed?
   d. Other?
4. For which purpose(s) would you consider using the WRASQ(L) (***select all that apply***):
   a. To document the occupational history and history of workplace exposures?
   b. To collect information about the relationship between your patients asthma symptoms and workplace exposures?
   c. To initiate a conversation around the topic of workplace-symptom relationship with patients?
   d. As a screening tool for WRA?
   e. As guide for next steps in WRA investigation?
   f. Other?
5. What would be your preferred method of distribution of the WRASQ(L)™?
   a. Paper
   b. Electronic
      i. Via kiosks/tablets that patients fill out before/during visit?
      ii. Within the EMR
   c. Either paper or electronic
   d. Other _________
6. Would you recommend the WRASQ(L)™ to patients/other providers if made available publicly online, such as in association websites, dashboards etc.? (***Yes,No,N/A ,other & space for comment***)
